# Species replacement dominates megabenthos beta diversity in a remote seamount setting

**DOI:** 10.1038/s41598-018-22296-8

**Published:** 2018-03-07

**Authors:** Lissette Victorero, Katleen Robert, Laura F. Robinson, Michelle L. Taylor, Veerle A. I. Huvenne

**Affiliations:** 1 National Oceanography Centre, University of Southampton Waterfront Campus, Southampton, SO14 3ZH United Kingdom; 2 University of Southampton, Ocean and Earth Science, Southampton, SO14 3ZH United Kingdom; 3 Fisheries and Marine Institute of Memorial University, St. John’s, NL A1C 5R3 Canada; 4 University of Bristol, School of Earth Sciences, Bristol, BS8 1RJ United Kingdom; 5 University of Essex, School of Biological Sciences, Colchester, CO4 3SQ United Kingdom

**Keywords:** Biodiversity, Marine biology

## Abstract

Seamounts are proposed to be hotspots of deep-sea biodiversity, a pattern potentially arising from increased productivity in a heterogeneous landscape leading to either high species co-existence or species turnover (beta diversity). However, studies on individual seamounts remain rare, hindering our understanding of the underlying causes of local changes in beta diversity. Here, we investigated processes behind beta diversity using ROV video, coupled with oceanographic and quantitative terrain parameters, over a depth gradient in Annan Seamount, Equatorial Atlantic. By applying recently developed beta diversity analyses, we identified ecologically unique sites and distinguished between two beta diversity processes: species replacement and changes in species richness. The total beta diversity was high with an index of 0.92 out of 1 and was dominated by species replacement (68%). Species replacement was affected by depth-related variables, including temperature and water mass in addition to the aspect and local elevation of the seabed. In contrast, changes in species richness component were affected only by the water mass. Water mass, along with substrate also affected differences in species abundance. This study identified, for the first time on seamount megabenthos, the different beta diversity components and drivers, which can contribute towards understanding and protecting regional deep-sea biodiversity.

## Introduction

Seamounts are prominent, globally distributed, isolated features, rising a minimum of 100 m from the surrounding seafloor^1^. Estimates suggest that there are ~33,000^2^ to ~100,000 such features worldwide^3^, but <4% of seamounts have been sampled for scientific purposes^4^. Seamounts are an important deep-sea habitat type^5^ since they host diverse benthic communities^6^ and can host abundant fish stocks^7^. The latter factor makes seamounts prominent targets for deep-sea bottom trawling^8^. Moreover, at present some seamounts covered with ferromanganese crusts are being considered as a potential source of cobalt, tellurium, and other valuable metals for mining^9^. Both these activities involve immense physical disruption through removal of bottom habitat and habitat-forming species in an ecosystem defined by slow-growing, long-lived organisms^10,11^. The impacts on the benthos are likely to be long-lasting or even permanent, as observed in the wake of bottom trawling fisheries on seamounts off New Zealand, where there has been no recovery over decadal time scales^12^. Such continuing interest to exploit deep-sea ecosystems provides a challenge for sustainable management^13^, which should be underpinned by knowledge on the spatial distribution of seamount biodiversity and its environmental drivers.

Seamounts, however, are not one single entity, as they have varied morphologies, depths and locations with consequently different local environments^14^, which results in different faunal compositions across seamounts^15^. At a broad regional scale, seamount benthic communities appear to be influenced by latitudinal turnover^16^, oxygen concentration^17^, aragonite saturation for stony corals^18^ and primary productivity. Seamount morphology might also play a role in controlling local particulate organic carbon (POC) export, as it has been shown that a tall seamount increased the POC export due to its interaction with tidal dynamics^19^. Furthermore, seamount seabed morphology leads to complex habitat mosaics at different depths that modify the hydrodynamic flow^20,21^. This diverse geomorphology coupled with the occurrence of habitat-forming species, causes high habitat heterogeneity, increases local (alpha) diversity^22^ and affects assemblage patterns through provision of a physical habitat for other invertebrates^23^. To date, seamount research has had a strong focus on comparing different sites, so there are only a handful of studies focusing on changes in megafaunal beta diversity (spatial differentiation of diversity) on single seamounts^24–27^, hindering our understanding of how species are partitioned amongst habitats. This knowledge is crucial for linking alpha and gamma (regional) diversity, leading to improved understanding of large-scale biogeographical gradients on seamounts, and more generally, within the deep-sea.

Deep-sea research has shown that depth differences result in greater beta diversity than horizontal distances^28,29^. Accordingly, previous research on the megafauna of Davidson Seamount found that depth influences changes in assemblage structure^30,31^, but does not affect alpha diversity or species density^30^. Conversely, in the off-shore Taney Seamount Chain, slope affected both beta diversity and density while depth affected alpha- and beta diversity^26^. The disparities in diversity patterns at different seamounts have been proposed to be driven by variations in surface productivity and depth gradients^26^. To build a complete understanding of spatial patterns on seamounts, however, it is beneficial to split beta diversity into components. This approach involves understanding which sites and species contribute to beta diversity, followed by the untangling of beta diversity into distinct processes, such as replacement (species replace each other along ecological gradients) and richness difference (different communities have a different number of species)^32,33^. This detailed information will allow for the understanding of the ecological processes behind each component and, subsequently, how these are spatially shaping the community assemblages.

Here we apply recently developed beta diversity analyses to benthic megafaunal communities in order to increase our comprehension of beta diversity patterns on seamounts. This study uses a multidisciplinary data set of Remotely Operated Vehicle (ROV) transects, bathymetry derived environmental variables, and oceanographic information to illustrate how beta diversity changes across a depth gradient and how different areas and species affect beta diversity. Furthermore, we show for the first time in a seamount benthos, the influence of an array of environmental variables on two distinct beta diversity processes: species replacement and species richness difference.

## Methodology

### Study site

**Figure 1:**
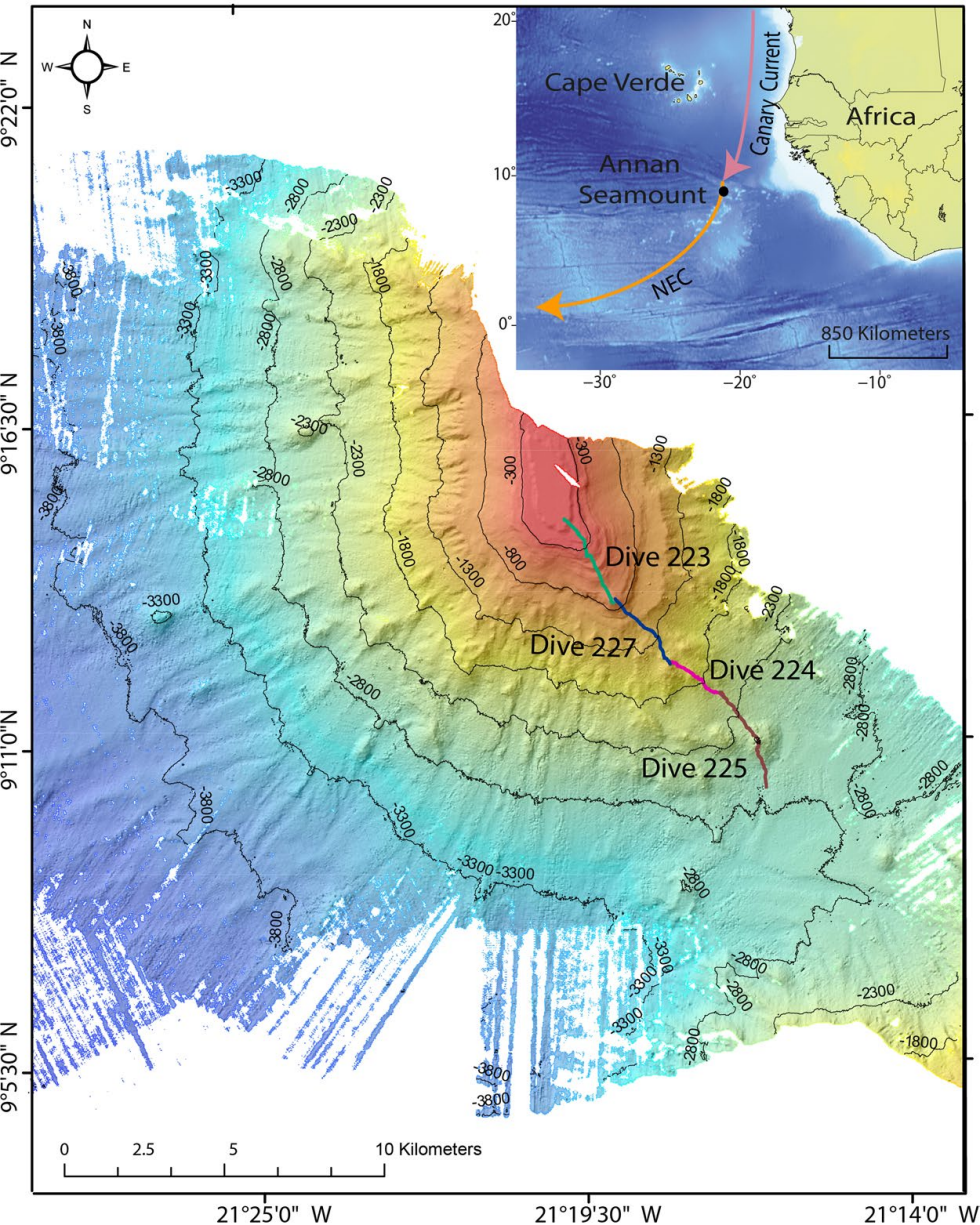
Map of the south-west section of Annan Seamount showing the location of the ROV tracks with bathymetry of 25 m pixel resolution. NEC = North-Equatorial Current. The site map was created in ESRI ArcMap (version 10.2.2). The insert map is ETOPO1 relief data created using Generic Mapping Tools^66^.

Annan Seamount, in the Equatorial-Atlantic, is part of the Grimaldi Bathymetrists Seamount Chain^34^, where it lies at depths of 200–4500 m (Fig. 1). Previous publications from the same research expedition have also referred to this feature as Carter Seamount. It is a conical guyot occurring at the northern termination of the large volcanic edifice, formed by the Sierra Leone Rise, with the rest of the topography consisting of long-offset fracture zones^34,35^. Annan Seamount has been hypothesised to have formed during the Palaeocene ~54.4 ± 1.1 Ma^34^. The seamount is bathed by three water masses, North-Atlantic Deep Water (NADW) at the base and slope up to ~2500 m depth, Antarctic Intermediate Water (AAIW) covering the flank up to ~1500 m, and South–Atlantic Central Water (SACW) to the summit^36^. Annan Seamount is affected by two current systems as it lies within the transition zone between the colder Canary Current flowing southwest and the warmer North Equatorial Current flowing east to west^37,38^ (Fig. 1). To date, little is known about the benthic communities of Annan seamount with this study presenting its first biological characterisation.

### Data collection and processing

**Figure 2:**
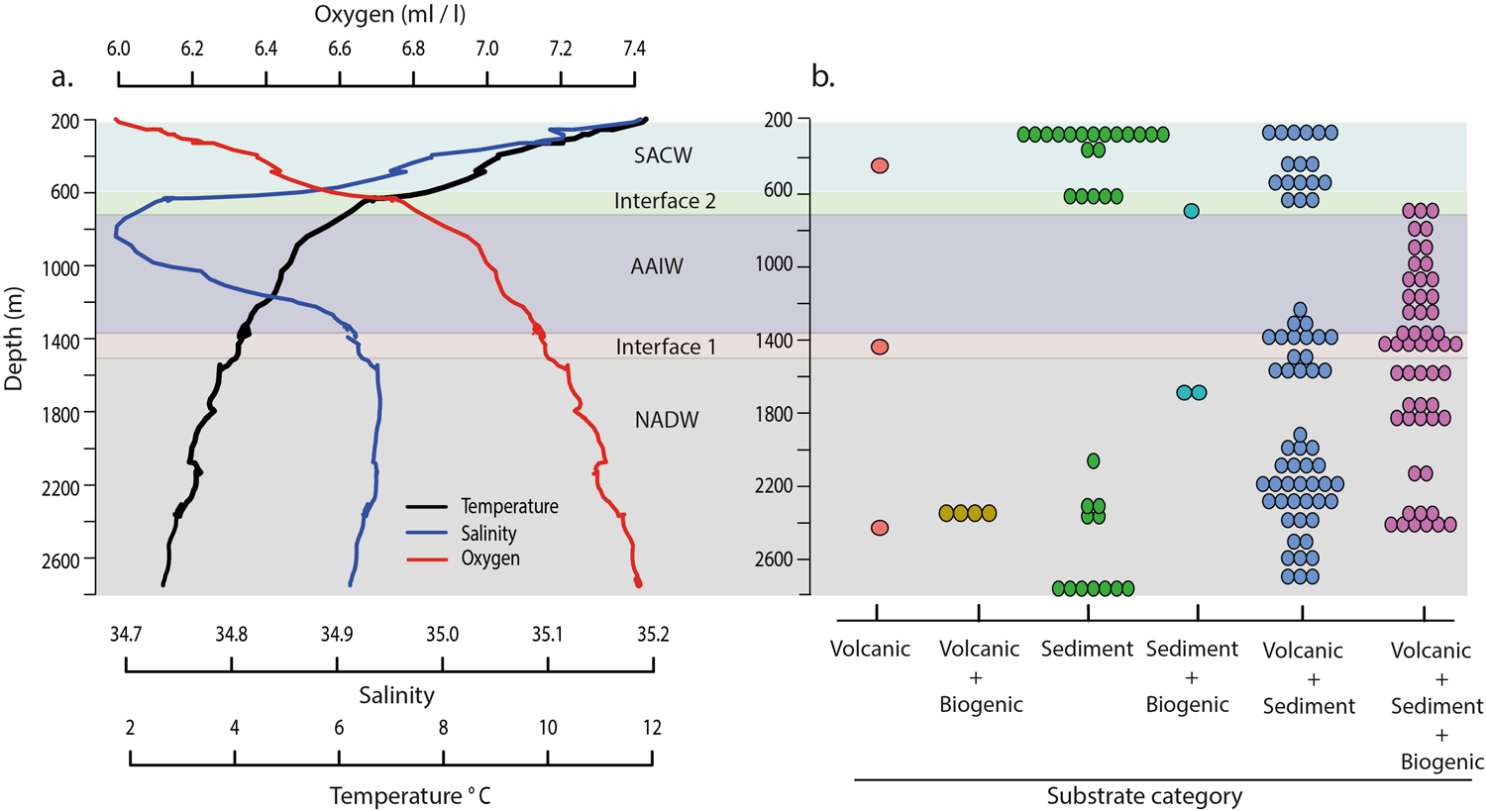
Temperature, salinity, oxygen and water mass categories from the ROV CTD (**a**) along with the different substrate categories across the seamount from the 100 m sample size (**b**). SACW = South-Atlantic Central Water, AAIW = Antarctic Intermediate Water, NADW = North- Atlantic Deep Water. The interfaces are defined from raw ROV CTD data (Supplementary Table S2).

The data for this study were collected on board the RRS *James Cook* in 2013 as part of the ERC funded Tracing Oceanic Processes using Corals and Sediments programme (TROPICS)^39^. The ROV *Isis* was used to collect video footage at Annan Seamount over four dives from depths of 200–2730 m, covering 11 km in distance and generating a total of 87 h of video material (Supplementary Table S1). The video material was used to identify and count benthic fauna. It was also used to annotate and classify substrates into six categories; (1) volcanic, (2) volcanic and biogenic, (3) sediment, (4) sediment and biogenic, (5) volcanic and sediment, (6) volcanic, sediment and biogenic (Fig. 2) (for examples of each category see Supplementary Fig. S1). A comprehensive description of the data processing steps related to the ROV imagery is available in Supplementary Material S1.1. The ROV was equipped with a SeaBird SBE 49 CTD that measured temperature, conductivity and pressure. All CTD data were processed with the SBE Data Processing (V7.20 g) software, which also calculated depth, salinity and theoretical oxygen saturation (hereafter referred to as oxygen). The ROV CTD data were also used to characterize the water masses *in situ* using the classification by Emery^40^.

Shipborne multibeam bathymetry data were acquired with the ship’s hull-mounted Kongsberg EM-120 echosounder and processed using CARIS HIPS and SIPS (v. 8.1). The data were gridded at 25 m resolution. The bathymetry was used for extracting quantitative descriptors of the seabed terrain^41^ (see Supplementary Material S1.2 for detailed description). These included slope, topographic position index (TPI) at two scales (3 × 3 and 9 × 9 pixels neighbourhood radius), roughness, aspect (divided in eastness and northness), and three measures of curvature (general, profile and plan)^42,43^. The species, substrate, temperature, salinity and oxygen, and bathymetry-derived data were then combined by spatial joins in ArcGIS (version 10.2.2). After evaluations of different sample sizes, it was decided to use 100 m and 200 m ROV transect sample lengths, which generated 160 samples and 78 samples, respectively. The finer scale sample size of 100 m was chosen on the basis of maintaining substrate and habitat fidelity whilst capturing enough individuals per sample to be able to perform community analyses. The broader scale of 200 m was used to investigate how strongly spatial scale affected beta diversity patterns and corresponding environmental drivers.

### Statistical analysis

Beta diversity analyses were conducted according to the methodology proposed by Legendre & De Cáceres^33^ and Legendre^44^ using the R package ‘adespatial’. These approaches allow for (i) no prior knowledge of the study area’s alpha or gamma diversity to estimate beta diversity, (ii) assessment of individual species’ and sample contribution to beta diversity and (iii) partitioning of the spatial variation and testing of explanatory environmental factors. Prior to the analysis, all species occurring only once throughout the study site were removed because (i) the nature of the data collection did not enable verification of their taxonomic identity and (ii) they were not deemed to be representatives of the community. Both presence – absence and species abundance data were generated. The former is suitable for the wide depth range covered in this study, while the latter provides an insight to differences in abundance. The data were transformed prior to statistical analyses using the Hellinger transformation, which limits the importance of rare taxa when computing dissimilarities as our survey was a depth transect (i.e. every depth was visited once), which could lead to the generation of false rare species.

The relative Local Contribution to Beta Diversity (LCBD) of each 100 m length sample and the Species Contribution to Beta Diversity (SCBD) were calculated using the function *beta*.*div*^33^. The LCBD represents the ecological comparative uniqueness of the sample, and its significance for each sample was assessed through a permutation analysis (p < 0.05, 999 iterations) testing the null hypothesis that species’ distribution is random among sampling units. The SCBD represents the degree of contribution of each species to the overall beta diversity and was calculated on the Hellinger-transformed species matrix.

In order to further understand the ecological processes behind beta diversity, a total beta diversity index was calculated. Total beta diversity was then partitioned into species replacement and richness difference by the *beta*.*div*.*comp* function using the Jaccard dissimilarity for presence-absence and species abundance data for abundance difference^44^. This computation provides indices for total beta diversity, species richness/abundance difference and replacement in addition to distance matrices. These indices determine the dominant process along the spatial gradient, while the matrices can be used to understand how environmental variables affect different components of beta diversity. For this, each matrix was used in a Principal Coordinates Analysis (PCoA) with a Lingoes correction to account for negative eigenvalues. The resulting coordinates, along with the environmental variables, were used as input in forward selection (*forward*.*sel* function) from which a set of estimated significant environmental variables were used as input explanatory variables in a Distance-based redundancy analysis (dbRDA). The absolute significance of the environmental variables behind the processes depicted by the Jaccard dissimilarity, richness difference and replacement matrices, was assessed with an F-test testing for significance (p < 0.05, 999 permutations)^44^. Several reiterations of the analysis were conducted in order to avoid correlation between co-variable oceanographic parameters (depth, temperature and oxygen) and terrain variables (broad and fine scale TPI and general, planar and profile curvature). The datasets generated during and/or analysed during the current study are available from the corresponding author on reasonable request.

## Results

### Overview of Annan Seamount biological communities

Annan Seamount was characterised by abundant and diverse megafaunal communities, generating a record of ~30 000 individual organisms separated into 180 morphospecies. After excluding species with only one occurrence, for reasons previously discussed, 150 morphospecies were used in subsequent analysis. Changes in community composition were apparent across the depth transect and different substrate types, with the following five distinct biological assemblages described in more detail. The summit of Annan at ~200 m depth was occupied by mobile benthic communities, mainly the ophiuroid *Ophiotreta valenciennesi*, an unknown white urchin species and the holothurian *Stichopotidae* sp. Below the summit, with increasing depth up to 650 m, the community was dominated by large aggregations of the urchin *Cidaris cidaris*, different species of anemones and the cushion-star *Plinthaster* sp. The depths of 1300–1500 m harboured extensive multi-species cold-water coral gardens, comprised of species such as *Paragorgia* sp., *Paramuricea* sp. and *Corallium* sp. along with unidentified primnoid and bamboo coral species and associated invertebrates. Several vertical walls and extremely steep slopes were observed, which were also colonized by similar morphospecies of coral, but their respective abundances varied. The deeper part of the study area, from ~2300 m onwards, was characterized by sediment plains interspersed by volcanic substrate and dead bamboo coral skeletons. These skeletons occurred on an elevated seabed and were occupied by high abundances of *Anthomastus* sp. (~3300 individuals in 1.5 km distance). The sediment plains down to ~2730 m depth were inhabited by a diverse holothurian community, with different species of the *Benthodytes* genus and the Synallactidae family along with *Peniagone* sp. and an unusual morphotype of the *Ellipinion* sp.

### Samples and species contribution to beta diversity

**Figure 3:**
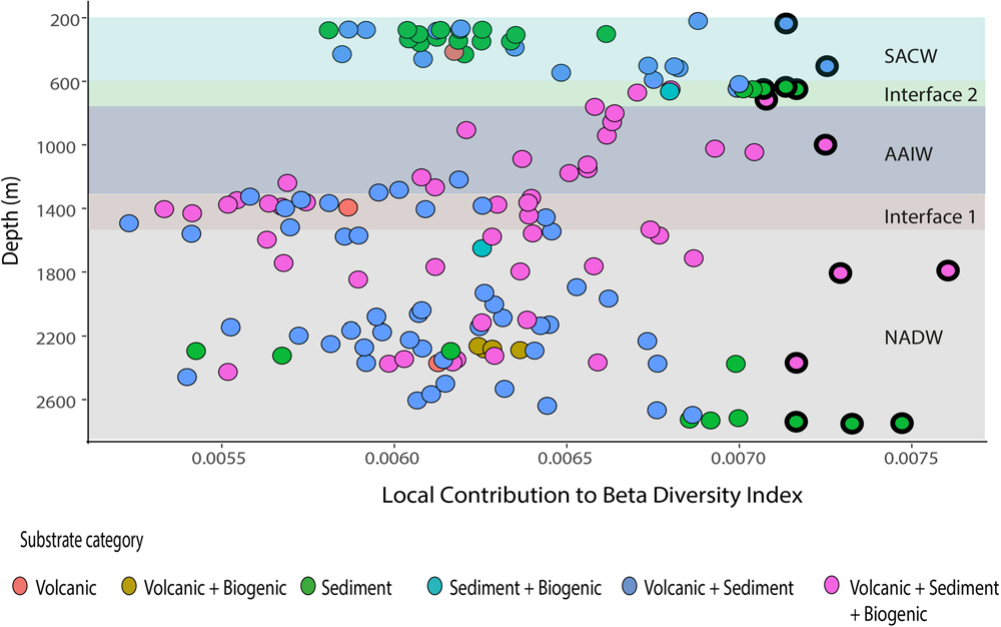
The Local Contribution to Beta Diversity Index of the 100 m length samples per substrate and water mass type. The black circles illustrate the significant samples at p < 0.05, n = 160.

**Figure 4:**
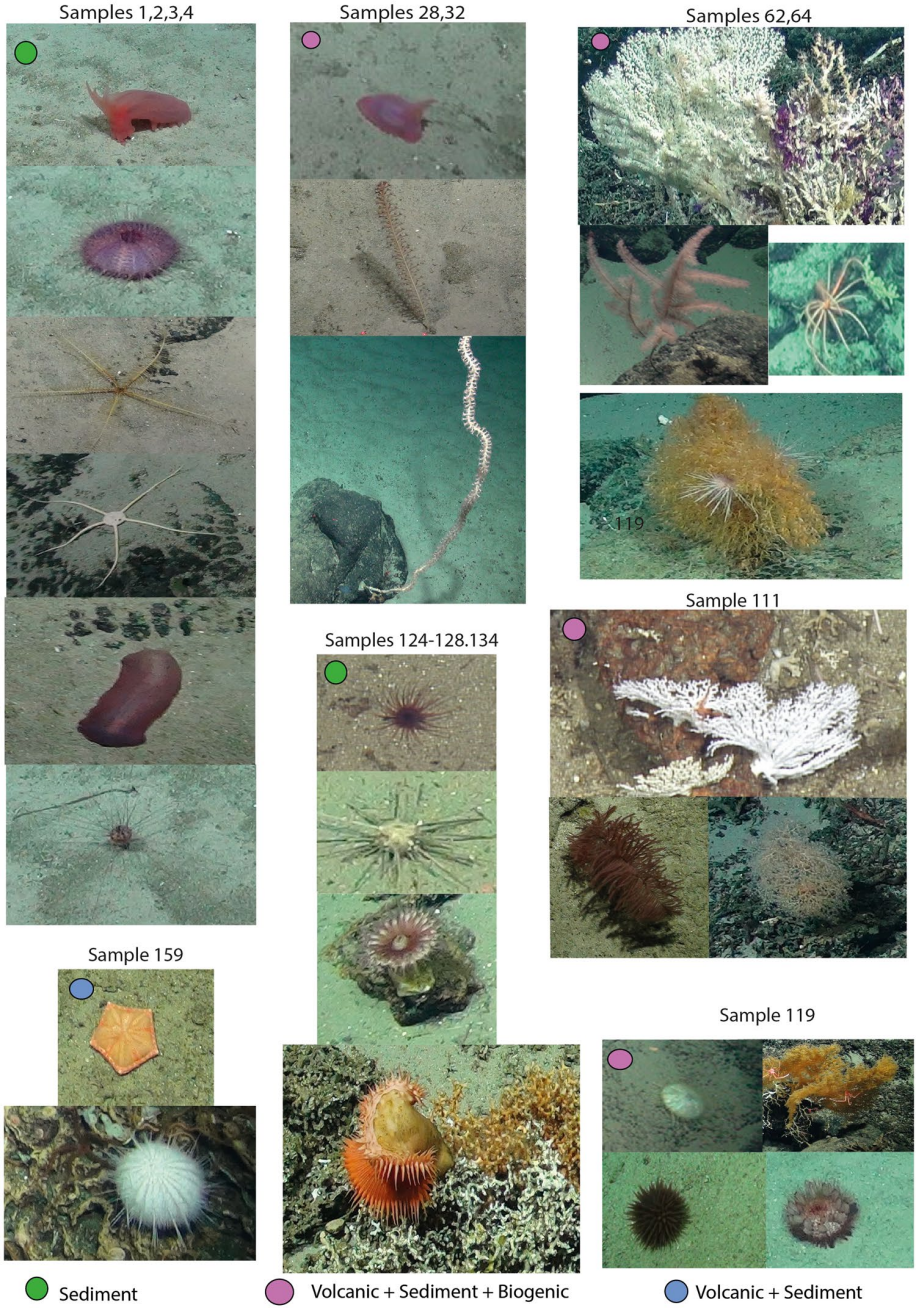
Examples of the communities of the significant Local Contribution to Beta Diversity samples with their corresponding habitats. The species names are per sample grouping from left to right and then from top to bottom. Samples 1,2,3,4 - *Peniagone or Amperima* sp., Pink echinothurioid, Comatulidae, *Ophiomusium* sp., *Benthodytes* sp. and *Aspidodiadema* sp.; samples 28, 32 - *Ellipinion* sp., *Funniculina* sp., Isididae whip; samples 62,65 - *Corallium* sp. and *Clavularia* sp., *Parantipathes* sp., Brisingidae, *Acanella* sp.; sample 111 - Stylasterid, *Bathypathes* sp. and Chrysogorgid.; sample 119 - white echinohurioid, *Leiopathes* sp., *Liponema* sp., *Phormosoma* sp.; samples 124–128,134 - brown cerianthid anemone, *Cidaris cidaris*, purple cerianthid anemone, Hormathiidae on *Solenosmilia variabilis*; sample 159 - *Plinthaster* sp. and Urchin white sp.2.

**Figure 5:**
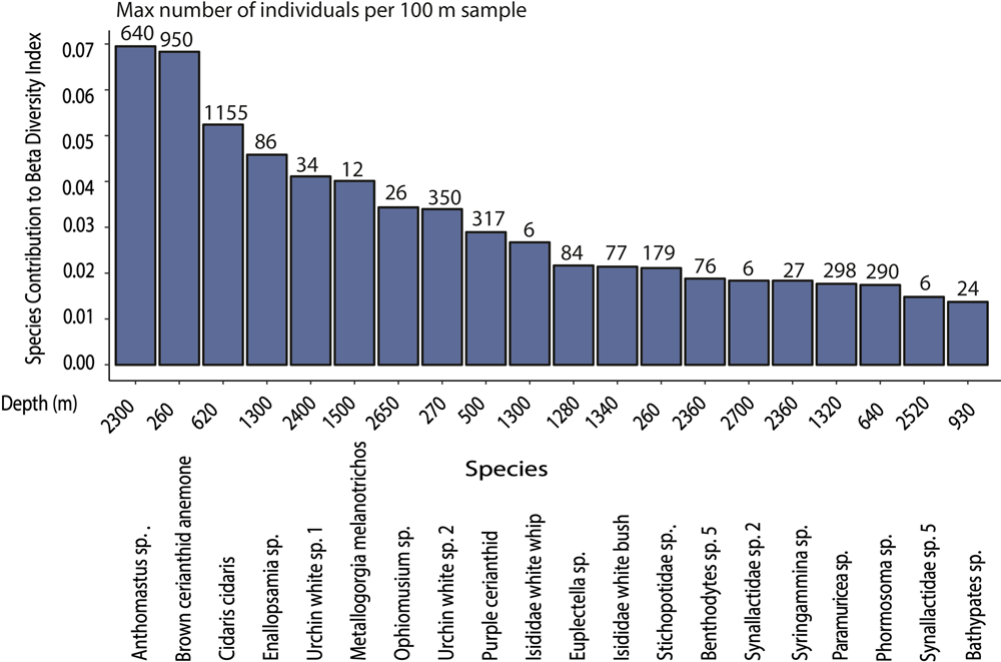
The contribution of the top 20 individual species to beta diversity and the maximum number of individuals per 100 m sample and corresponding depth.

The total beta diversity index for Annan Seamount was high, with the value of 0.92 out of the maximum possible value of 1, which occurs when all sites contain different species. The values of the contribution of individuals samples to beta diversity, as calculated from the LCBD analysis, ranged from 0.0009 to 0.007 (Fig. 3). The p-values for the significantly higher beta diversity samples ranged from 0.001 to 0.005. At the shallower sites within the SACW, the ecologically unique samples were on mixed substrates (Fig. 3), and consisted of feeding aggregations of the asteroid *Plinthaster sp*., an unknown species of urchin (Urchin white sp.2) and *Ophiotreta valenciennesi* (Figs 3 and 4). The latter two species also exhibited large variance across the study site as depicted by the high SCBD values (Fig. 5). The significant communities at the interface between SACW and AAIW were sediment communities highly dominated by cerianthid anemones and *C*. *cidaris* (Fig. 3), with the latter, due to urchin aggregations, having some of the highest SCBD values across this study (Fig. 5). Within the AAIW, and the upper part of the NADW, the significant LCBD sites were highly heterogenous habitats (Fig. 3), hosting urchins on the sediment substrates, and corals and associated fauna attached to dead coral skeletons and large boulders (Fig. 4). The significant samples at the deeper part of the NADW were sediment communities (Fig. 3), but contrary to shallow sediment habitats, the distribution of the different species of echinoids and holothurians, such as *Scotoplanes* (seapigs) and *Benthodytes* sp., was mostly sparse. Conversely, *Anthomastus sp*., which had the overall highest SCBD value, also occurred within the depths of the NADW in large aggregations over 100 m distances (Fig. 5). Other species with high SCBD values were *Enallopsammia* sp. and *Metallogorgia melanotrichos* (Fig. 5). Specifically, *M*. *melanotrichos* was scattered, contributing to a variety of different communities in low abundances throughout the study site.

### Partitioning of total beta diversity

**Figure 6:**
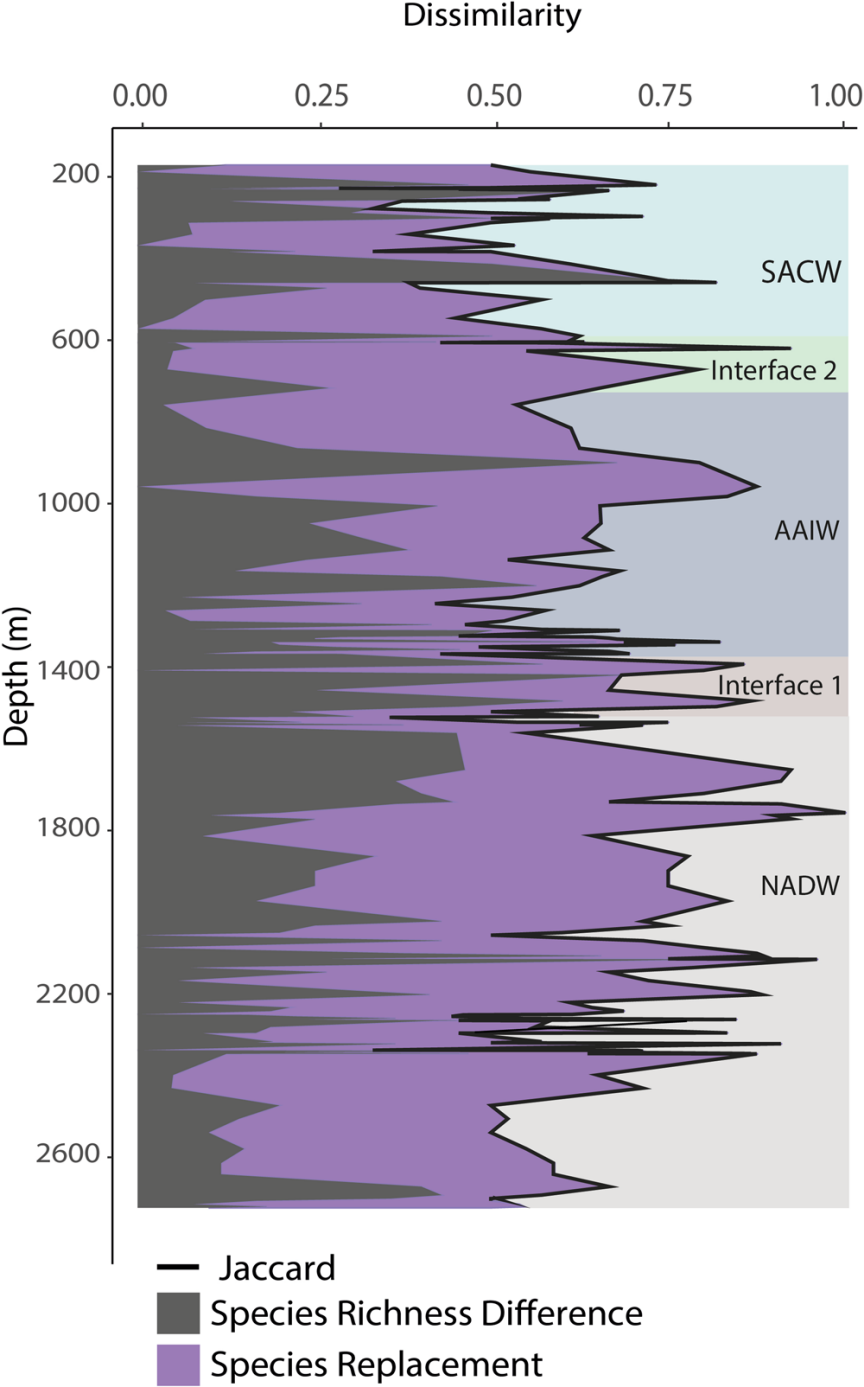
The Jaccard based dissimilarity using presence – absence data between each consecutive 100 m length sample showing the relative proportion of species replacement *vs*. species richness difference contribution towards total beta diversity across the depth range. SACW = South-Atlantic Central Water, AAIW = Antarctic Intermediate Water, NADW = North-Atlantic Deep Water. The interfaces are defined from raw ROV CTD data (Supplementary Table S2).

The dissimilarities, in the form of Jaccard dissimilarity, species replacement and richness difference, between samples were extremely high throughout the study transect (Fig. 6). These patterns were similar at both sampling scales as well as for both presence-absence and species abundance data. Partitioning of the total beta diversity, using presence-absence data, showed the species replacement component to be dominant (~68%), with the species richness difference contributing only ~32%. The species richness difference component was more dominant at ~200 and ~500 m water depth (Fig. 6). It was also relatively high at ~1300–1500 m depth, albeit fluctuating rapidly with the species replacement component (Fig. 6).

The significant variables explaining the patterns in the Jaccard dissimilarity matrix were water depth and correlated variables (oxygen and temperature), water mass, substrate, slope, eastness and northness (aspects), and broad scale TPI (R^2^_adj_ = 0.24) (Table 1). It is worth noting that temperature, depth and oxygen had the same p and R^2^_adj_ - values for this matrix. For the species replacement matrix the significant variables with the highest R^2^_adj_ (0.24) were depth, water mass, broad scale TPI and northness (Table 1). The species richness difference component was explained purely by water mass in the presence/absence data (R^2^_adj_ = 0.14). Water mass also affected the species abundance difference, along with substrate (R^2^_adj_ = 0.27) (Table 1.). At both scales the explanatory variables were similar and measures of curvature and surface roughness were not found to influence the different components of beta diversity.

**Table 1:** Results from the Distance-based redundancy analysis (dbRDA) with a forward selection, showing the significant environmental variables (p < 0.05) affecting the Jaccard dissimilarity, species replacement, species richness/abundance difference using (a) presence –absence and (b) species abundance data of 100 m length samples (n = 160).

	R^2^_adj_		Water mass	Substrate	Slope	Eastness	Northness	Broad-scale TPI
**a) Presence-absence**								
Jaccard_100m_	0.22	Depth *p* = *0*.*001*	*p* = *0*.*001*	*p* = *0*.*002*	*p* = *0*.*001*	*p* = *0*.*004*	*p* = *0*.*015*	*p* = *0*.*001*
	0.22	Temperature *p* = *0*.*001*	*p* = *0*.*001*	*p* = *0*.*001*	*p* = *0*.*003*	*p* = *0*.*012*	*p* = *0*.*003*	*p* = *0*.*001*
	0.22	Oxygen *p* = *0*.*001*	*p* = *0*.*001*	*p* = *0*.*001*	*p* = *0*.*008*	*p* = *0*.*008*	*p* = *0*.*011*	*p* = *0*.*001*
Repl_100m_	0.24	Depth *p* = *0*.*001*	*p* = *0*.*001*	*not sig*.	*not sig*.	*not sig*.	*p* = *0*.*007*	*p* = *0*.*001*
	0.22	Temperature *p* = *0*.*001*	*p* = *0*.*001*	*p* = *0*.*003*	*p* = *0*.*003*	*p* = *0*.*003*	*p* = *0*.*010*	*p* = *0*.*003*
	0.24	Oxygen *p* = *0*.*001*	*p* = *0*.*001*	*p* = *0*.*007*	*p* = *0*.*001*	*p* = *0*.*001*	*p* = *0*.*001*	*p* = *0*.*001*
Rich. diff._100m_	0.14	*not sig*.	*p* = *0*.*001*	*not sig*.	*not sig*.	*not sig*.	*not sig*.	*not sig*.
**b) Species abundance**								
Jaccard_100m_	0.20	Depth *p* = *0*.*001*	*p* = *0*.*001*	*p* = *0*.*001*	*p* = *0*.*001*	*p* = *0*.*001*	*p* = *0*.*018*	*p* = *0*.*010*
	0.20	Temperature *p* = *0*.*001*	*p* = *0*.*001*	*p* = *0*.*001*	*p* = *0*.*001*	*p* = *0*.*001*	*p* = *0*.*014*	*p* = *0*.*001*
	0.20	Oxygen *p* = *0*.*001*	*p* = *0*.*001*	*p* = *0*.*002*	*p* = *0*.*001*	*p* = *0*.*002*	*p* = *0*.*018*	*p* = *0*.*001*
Repl_100m_	0.19	Depth *p* = *0*.*001*	*p* = *0*.*001*	*p* = *0*.*002*	*p* = *0*.*001*	*p* = *0*.*001*	*p* = *0*.*001*	*p* = *0*.*001*
	0.19	Temperature *p* = *0*.*001*	*p* = *0*.*001*	*p* = *0*.*011*	*p* = *0*.*002*	*p* = *0*.*003*	*p* = *0*.*010*	*p* = *0*.*010*
	0.19	Oxygen *p* = *0*.*001*	*p* = *0*.*001*	*p* = *0*.*008*	*p* = *0*.*001*	*p* = *0*.*001*	*p* = *0*.*001*	*p* = *0*.*001*
Abun. diff._100m_	0.27	*not sig*.	*p* = *0*.*001*	*0*.*042*	*not sig*.	*not sig*.	*not sig*.	*not sig*.

## Discussion

This study applied recently developed beta diversity landscape metrics to a seamount benthos across a bathymetric gradient. The analysis identified sites with ecologically unique communities (LCBD), and species which showed high variance across the seamount (SCBD), thus contributing to high beta diversity. The decomposition of beta diversity, quantified, for the first time in seamount benthos, the respective proportions of species replacement and species richness difference. Furthermore, this decomposition allowed us to assess the effects of *in situ* depth related parameters obtained by the CTD on the ROV, and relatively fine scale terrain variables, on each beta diversity mechanism.

Total beta diversity at Annan Seamount was high in this study (0.92 out of 1), showing that community level turnover along the depth transect was frequent. In comparison, the macrobenthos in the coastal areas of the Red Sea exhibits an index of 0.43^45^, while the macrofauna and meiofauna in hydrothermal vents only exhibit ~0.36^46^ of the maximum total beta diversity of 1. The significant LCBD samples above ~600 m depth, had highly dominant species present, which also had large SCBD values, such as *Cidaris cidaris* (Fig. 5). While this urchin occurs on dead coral substrates in addition to sediments, *C*. *cidaris* was found to form large aggregations in soft sediments, which were flagged as significant LCBD sites (Fig. 2). Histological studies on their gonads revealed that the urchins were mature and aggregating to spawn^47^. Previous observations of *C*. *cidaris* aggregations suggested that these are for both reproductive and feeding purposes and form irrespective of the presence of predators^48^. This detailed information available on one species highlights that temporal biological interactions are likely to be important in driving local beta diversity by producing ecologically unique communities.

The majority of the species contributing towards beta diversity on Annan Seamount had patchy distributions, often characterised by high abundance over a limited depth range resulting in high variance (Fig. 5). Conversely, a previous study in Taney Seamount attributed faunal turnover to the rarer species, which were suspected to have a narrower depth range while abundant fauna would be expected to have a wider depth range^26^. The contrasting results might arise from different methodological approaches as McClain and Lundsten^26^ applied a log- transformation for data normalisation, giving more importance to rare taxa. Differences could also arise from seamounts being variable in terms of their morphology and summit height, which in turn affects productivity, potentially leading to ecologically distinct beta diversity patterns^26,49^. However, results from streams and lakes support our findings, by showing that abundant species occupying an intermediate number of sites contribute most to beta diversity^50,51^. The abundant fauna in the present study were often confined to an optimal environmental niche, where they thrived, dominating their respective communities causing visual zonation patterns. A compressed depth range for megafauna has been previously linked to intrinsic mechanisms, such as competition for smaller prey, opportunistic species outcompeting others, or the local availability of food parcels^52,53^.

Partitioning of beta diversity on Annan Seamount shows that it is dominated by species replacement (68%), with differences in species richness only accounting for 32% of the total beta diversity (0.92). A dominating replacement component along a depth-gradient has also been found in asteroids and holothurians in the Porcupine Seabight and Porcupine Abyssal Plain^54^. The majority of the explanatory variables used in this study were correlated with changes in the different beta diversity components. Dissimilarity between samples was influenced by the oceanographic variables, depth, temperature and oxygen, the factorial variables of water mass and substrate, in addition to terrain properties such as slope, aspect and TPI. Here we are able to pinpoint that depth and correlated variables have an impact on beta diversity specifically through the species replacement component, likely by creating physiological niches across the seamount. It must be noted, however, that our data set does not contain any replicate transects and different patterns could be present in other locations on the seamount.

Water depth, which is also a proxy for other parameters, influencing community structure is a well-documented phenomena in seamount benthos^26,30^. It is thought to represent energy availability in the form of temperature and POC flux to the deep-sea^29^. Previous studies on bivalves across depth transects show that replacement is more dominant in productive regions. In lower productivity areas, nestedness, which is a type of species richness difference leading to ordered species loss, prevails^55^. Therefore, high species replacement in Annan Seamount could arise from its proximity to a relatively productive region off the west-African coast^56^. Water masses also influence the species replacement component, perhaps by acting as barriers causing genetic divergence resulting in closely related species sorting into depth bands, as seen in octocorals^57^.

The species replacement component was also affected by bathymetric properties, such as TPI and the northness of the seabed. These are both variables that have a more localised effect on the hydrodynamical flow. The aspect of the seabed influences the direction of the hydrodynamical flow and as such changes in this variable could be reflected in the presence or absence of sessile suspension feeders. The direction of the flow could be particularly relevant for large communities of habitat-forming species, such as cold-water corals, which are known to prefer to grow perpendicular to the water flow in order to optimize food capture^58^. There are no *in situ* hydrodynamical flow data available, but the main orientation of the ROV dives was south-easterly. As such, northness being associated with the species replacement component, suggests that the analysis has successfully captured communities with differential flow preferences.

The other variable affecting species replacement was TPI, which provides information on whether an area is elevated or has low to no relief in comparison to the surrounding seabed morphology. As such, it also affects which species are present by providing elevation and thus access to currents, or alternatively, seabed depression to those species that require it. Furthermore, the community composition of species replacement, as assessed by species abundance data, was also influenced by slope. This means that slope affects the relative abundances of the species present and as such, the community dynamics. Such differences were also observed from the imagery data, as certain species, such as Isididae whip corals and flytrap anemones, were more abundant on vertical walls and ledges while others, such as *Corallium* sp., *Enallopsammia* sp. and *Paramuricea* sp. preferred more gently sloping sites. Similarly, previous research on Taney Seamount Chain found that species are likely to favor horizontal or vertical surfaces as both extremes had higher faunal density than intermediate slopes^26^.

Perhaps surprisingly, none of the individual oceanographic variables linked with the richness difference component of beta diversity, but those variables combined, feed into the factorial water mass parameter. Water masses are a global scale variable with fluctuations in their characteristics over geological time scales. As such, the water mass variable is likely to affect larval transport to the seamount by acting as a connective pathway between deep-sea habitats^59^ as the dispersal range of deep-sea fauna is relatively large^60^. Additionally, water mass interfaces have also been proposed to enhance species richness by allowing the co-existence of warmer and colder-origin species^27^. The impact of water masses on the species richness difference component of beta diversity could also be linked to seamount fauna often reflecting the regional species pool^6,24,61,62^ with strong similarities with nearby banks^63^ and continental slopes^16^. The benthos of Annan Seamount is likely to be affected by all these mechanisms, as it lies between a cooler Canary Current and a warmer North-Equatorial Current system, and expands across three water-masses. Therefore, it is likely that other large scale variables, such as currents, along with evolutionary variables, affect the species richness difference component in marine systems.

Additionally, the use of species abundance data revealed that water masses along with substrate affect differences in abundances. As is the case with most seamounts, the substrates encountered were highly heterogeneous, encompassing hard firm volcanic rock, boulder fields, dead coral debris and mixed sediment types. It must be noted, however, that certain substrate types were markedly more common than others and, as such, there is a strong bias towards higher beta diversity indices in certain substrates due to under sampling of others (Fig. 2). Despite this, the effect of substrate on the species composition shows that while many of the species in the study occur over a multitude of substrate types, they are significantly more abundant in their preferred substrate type. For example, cold-water corals will grow over dead coral skeletons, rocks, boulders, pavements and outcrops as these all provide a firm attachment point and a degree of elevation into the currents. However, in this study, substantial multispecies coral gardens were seen where there were extensive volcanic plateaus available.

The effect of the substrate on replacement was also obvious at the summit, where zonation took place with ophiuroids dominating large encrusted hard surfaces, cerianthids the soft sediments, and an unknown species of white urchin occurring on top of the large boulders. Conversely, at depth, the soft-sediment communities were diverse and sparse, with a more gradual zonation of different species of holothurians, ophiuroids and xenophyophores, perhaps caused by small-scale sediment characteristics.

Overall, our results show that high habitat heterogeneity combined with the biological (or physiological?) effects of depth increases local biodiversity by providing neighboring areas with different ecological niches. This environmental complexity increases beta diversity and thus local biodiversity by affecting the species composition mostly through the replacement component and to a lesser degree by increasing or decreasing the number of species co-existing. While most of the measured variables showed a highly significant relationship with the beta diversity components, the explained variance remained relatively low throughout the study. This low variance intuitively suggests that these variables only represent a fraction of the environmental drivers behind changes in beta diversity and that noise was introduced to the data by studying a wide range of taxa. The low explanatory power of beta diversity models, however, is common across biomes and is thought to arise from the large influence randomness has on evolutionary and ecological processes^64^. Furthermore, the lack of coherent information on the basic biology and ecology of the deep-sea benthos^65^ makes understanding deep-sea beta diversity patterns challenging across local, regional and global scales. The large knowledge gap also results in difficulties predicting the consequences of anthropogenic activities and predicting recovery timelines^12^.

The landscape metrics applied in this study, however, provide a useful tool for understanding ecological patterns in the deep-sea. For example, species contributions to beta diversity are expected to change in response to anthropogenic disturbances^51^. As such, these indices could provide useful when monitoring and mitigating impacts from habitat fragmentation such as those potentially linked to deep-sea mining. In particular, this study suggests that in order to maintain high beta diversity on seamounts, species replacement and richness difference, should be considered as separate entities, as they appear to be affected, respectively, by local and global environmental parameters. Future work should concentrate on testing whether these patterns hold across wider deep-sea environments, comparing total beta diversity on different seamounts and testing beta diversity patterns of individual taxocenes. The results also indicate the necessity to incorporate quantitative descriptors of the seabed terrain along with large scale-variables, such as POC flux and ocean currents, to future beta diversity analyses.

## Conclusion

This study describes for the first time the biological communities of Annan Seamount. The seamount was characterized by diverse and abundant benthic megafauna, which were used to analyse beta diversity patterns using novel landscape metrics, never before applied to the seamount benthos. Ecologically important sites and communities were distinguished along with highly variant species, which contributed towards beta diversity. Changes in beta diversity are mostly dominated by species replacement with a smaller contribution from changes in species richness difference. Most importantly, this study confirms statistically, for the first time, how changes across an oceanographic gradient and local–scale terrain variables affecting flow regimes, impact beta diversity through species replacement on seamounts. Interestingly, the results demonstrate that beta diversity caused by changes in species richness can be linked with large-scale circulation patterns, which along with substrate, also causes changes in species abundance.

## Electronic supplementary material

Supplementary information
