# Author Correction: Species replacement dominates megabenthos beta diversity in a remote seamount setting

**DOI:** 10.1038/s41598-019-43802-6

**Published:** 2019-05-21

**Authors:** Lissette Victorero, Katleen Robert, Laura F. Robinson, Michelle L. Taylor, Veerle A. I. Huvenne

**Affiliations:** 1 National Oceanography Centre, University of Southampton Waterfront Campus, Southampton, SO14 3ZH United Kingdom; 2 University of Southampton, Ocean and Earth Science, Southampton, SO14 3ZH United Kingdom; 3 Fisheries and Marine Institute of Memorial University, St. John’s, NL A1C 5R3 Canada; 4 University of Bristol, School of Earth Sciences, Bristol, BS8 1RJ United Kingdom; 5 University of Essex, School of Biological Sciences, Colchester, CO4 3SQ United Kingdom

Correction to: *Scientific Reports* 10.1038/s41598-018-22296-8, published online 07 March 2018

The original version of this Article contained errors. Affiliations 1 and 3 were incorrectly given as ‘National Oceanography Centre, Southampton, SO14 3ZH, United Kingdom’ and ‘University of Oxford, Department of Zoology, Oxford, OX2 6GG, United Kingdom’ respectively. In addition, an affiliation was omitted, which is now listed as Affiliation 5.

Furthermore, the authors of this manuscript were incorrectly affiliated. Lissette Victorero was incorrectly affiliated with ‘National Oceanography Centre, Southampton, SO14 3ZH, United Kingdom’. Katleen Robert was incorrectly affiliated with ‘National Oceanography Centre, Southampton, SO14 3ZH, United Kingdom’. Laura F. Robinson was incorrectly affiliated with ‘University of Oxford, Department of Zoology, Oxford, OX2 6GG, United Kingdom’. Michelle L. Taylor was incorrectly affiliated with ‘University of Bristol, School of Earth Sciences, Bristol, BS8 1RJ, United Kingdom’. Veerle A.I. Huvenne was incorrectly affiliated with ‘University of Southampton, Ocean and Earth Science, Southampton, SO14 3ZH, United Kingdom’.

The correct affiliations list and affiliated authors are given below.

Affiliation 1:

National Oceanography Centre, University of Southampton Waterfront Campus, Southampton, SO14 3ZH, United Kingdom

Lissette Victorero

Katleen Robert

Veerle A.I. Huvenne

Affiliation 2:

University of Southampton, Ocean and Earth Science, Southampton, SO14 3ZH, United Kingdom

Lissette Victorero

Affiliation 3:

Fisheries and Marine Institute of Memorial University, St. John’s, NL A1C 5R3, Canada

Katleen Robert

Affiliation 4:

University of Bristol, School of Earth Sciences, Bristol, BS8 1RJ, United Kingdom

Laura F. Robinson

Affiliation 5:

University of Essex, School of Biological Sciences, Colchester, CO4 3SQ, United Kingdom

Michelle L. Taylor

These errors have now been corrected in the PDF and HTML versions of the paper, and in the accompanying Supplementary information file.

