# Supplementary material for: Species replacement dominates megabenthos beta diversity in a remote seamount setting
Source: Sci Rep. 2018 Mar 7;8:4152. doi: 10.1038/s41598-018-22296-8 (PMC5841424; doi:10.1038/s41598-018-22296-8)
Supplement: Supplementary file 1 — Supplementary information [file 41598_2018_22296_MOESM1_ESM.doc]

**Supplementary Information**

**Species replacement dominates megabenthos beta diversity in a remote seamount setting**

Lissette Victorero^1,2,*^ , Katleen Robert^1,3^, Laura F. Robinson^4^, Michelle L. Taylor^5^ and Veerle A.I. Huvenne^1^

**Data acquisition and processing**

S1.1 Data collection from Remotely Operated Vehicle (ROV) imagery

Video from the Super SCORPIO camera on the ROV was used for annotations. This camera was mounted on a fixed bracket on the starboard side of the centre line of the vehicle, had a laser harness (two lasers 0.1 m apart parallel to the focal axis), and a resolution of 1920 x 1080 HD. Two more cameras mounted on pan-and-tilt modules were used to support the identifications from the SCORPIO. Once on the seabed, cameras were white balanced to obtain optimal video quality. Video annotations were performed using Ocean Floor Observation Protocol (OFOP, v. 3.3.7a), which enables georeferenced faunal observations using the ROV’s ultra-short baseline (USBL) navigation system^1^. The ROV was also equipped with an altimeter and a depth sensor. To obtain statistical consistency all annotations were limited to sections where the ROV stayed within an altitude of 1-2.5 m above the seafloor; above this identifications became difficult. The minimum size of organisms recorded was ~3 cm. The video annotations were made by designating taxa to the highest taxonomic resolution possible based on visual assessment using species catalogues, the use of voucher specimens collected during the cruise, and consultation with taxonomic experts. The majority of identifications in this study are tentative. As such, the resulting morphospecies restrain the study and are likely to result in an underestimation of diversity as they do not account for cryptic species or smaller megafauna. This is, however, the most tractable methodology when using ROV imagery data, which is a widely used approach in deep-sea research^2^.Substrate was initially annotated using the habitat classification for seamount landscapes^3^, which resulted in three main substrate types with subclasses. The volcanic habitats, which included mainly rocky plateaus, walls, ledges and large boulders, the sediment habitats which included gravel, sand and ripples and the biogenic habitats, typified by dead coral skeletons. As these often occurred simultaneously in our study site, they were combined to make six substrate categories; 1) volcanic, 2) volcanic and biogenic, 3) sediment, 4) sediment and biogenic, 5) volcanic and sediment, 6) volcanic, sediment and biogenic (Fig.2) (for examples of each category see Supplementary Fig. S1). The substrate, species, and CTD data were then imported in to ArcGIS (version 10.2.2) for spatial analyses.

S1.2 Bathymetry data

When acquiring bathymetry using the EM-120 system, the swath width was around 3.5-5 times the water depth and the beam angles were kept around 60° - 65° port and starboard. The resulting bathymetry data was gridded at 25 m resolution to provide basemaps with the spatial reference of World Geodetic System 1984 Universal Transverse Mercator Zone 27 N. In ArcGIS, the Ultra-short baseline (USBL) track was cleaned and the inherent noise caused by USBL jumps smoothed using the Editing and Cartography Tools. Transects were first divided into 20 m long samples. The bathymetry-derived variables were generated using Jenness Enterprises’ Spatial Analyst Extension and Land Facet Corridor Tools ^4,5^ by calculating each variable from the 25 m pixel resolution basemaps and extracting its value for each sample’s midpoint. The samples were later scaled up to 100 m and 200 m sample lengths for reasons discussed in the main text.

**References**

1. Huetten, E. & Greinert, J. Software controlled guidance , recording and post-processing of seafloor observations by ROV and other towed devices : The software package OFOP. **10,** (2008).

2. Durden, J. M., Schoening, T. & Althaus, F. Perspectives in visual imaging for marine biology and ecology: from acquisition to understanding. *Oceanogr. Mar. Biol. An Annu. Rev.* (2016).

3. Auster, P. J., Moore, J. A. & Watling, L. A habitat classification scheme for seamount landscapes: assessing the functional role of deep-water corals as fish habitat Chapter. *Cold-Water Corals Ecosyst.* **10,** 605–621 (2005).

4. Jenness, J. DEM surface tools. (2012).

5. Jenness, J. Land facet corridor designer. (2012).

**Table S1. Information of the ROV dives used in the study**

| ROV ISIS Dive | Time spent at the seafloor (h) | Depth range (m) | Start of transect | End of transect |
| --- | --- | --- | --- | --- |
| 223 | 16.18 | 666 - 200 | 9° 13' 37" N  21° 18' 83" W | 9° 14' 82" N  21° 19' 71" W |
| 224 | 15.6 | 2132 -1350 | 9° 11' 73" N  21° 17' 05" W | 9° 12' 36" N  21° 17' 90" W |
| 225 | 19.38 | 2742 - 2100 | 9° 10' 17" N  21° 15' 92" W | 9° 11' 40" N  21° 16' 08" W |
| 227 | 18.73 | 1300 - 680 | 9° 12' 33" N  21° 17' 94" W | 9° 13' 45" N  21° 18' 81" W |

**Table S2. Ranges used for water mass and interface classification from the ROV CTD**

| Water mass | Depth (m) | Temperature (°C) | Salinity (ppt) |
| --- | --- | --- | --- |
| NADW | 2500 - bottom | 1.5-4 | 34.8-35 |
| Interface 1 | 1550-1320 | 4.1-4-4 | 35-34.9 |
| AAIW | 1500-750 | 2-6 | 33.8-34.8 |
| Interface 2 | 750-600 | 6-7.9 | 34.7-34.9 |
| SACW | 500 - surface | 5-18 | 34.3-35.8 |

*Core water mass parameters are per Emery (2003). Interfaces were defined when depth, temperature and salinity values were not all simultaneously within the core values.*


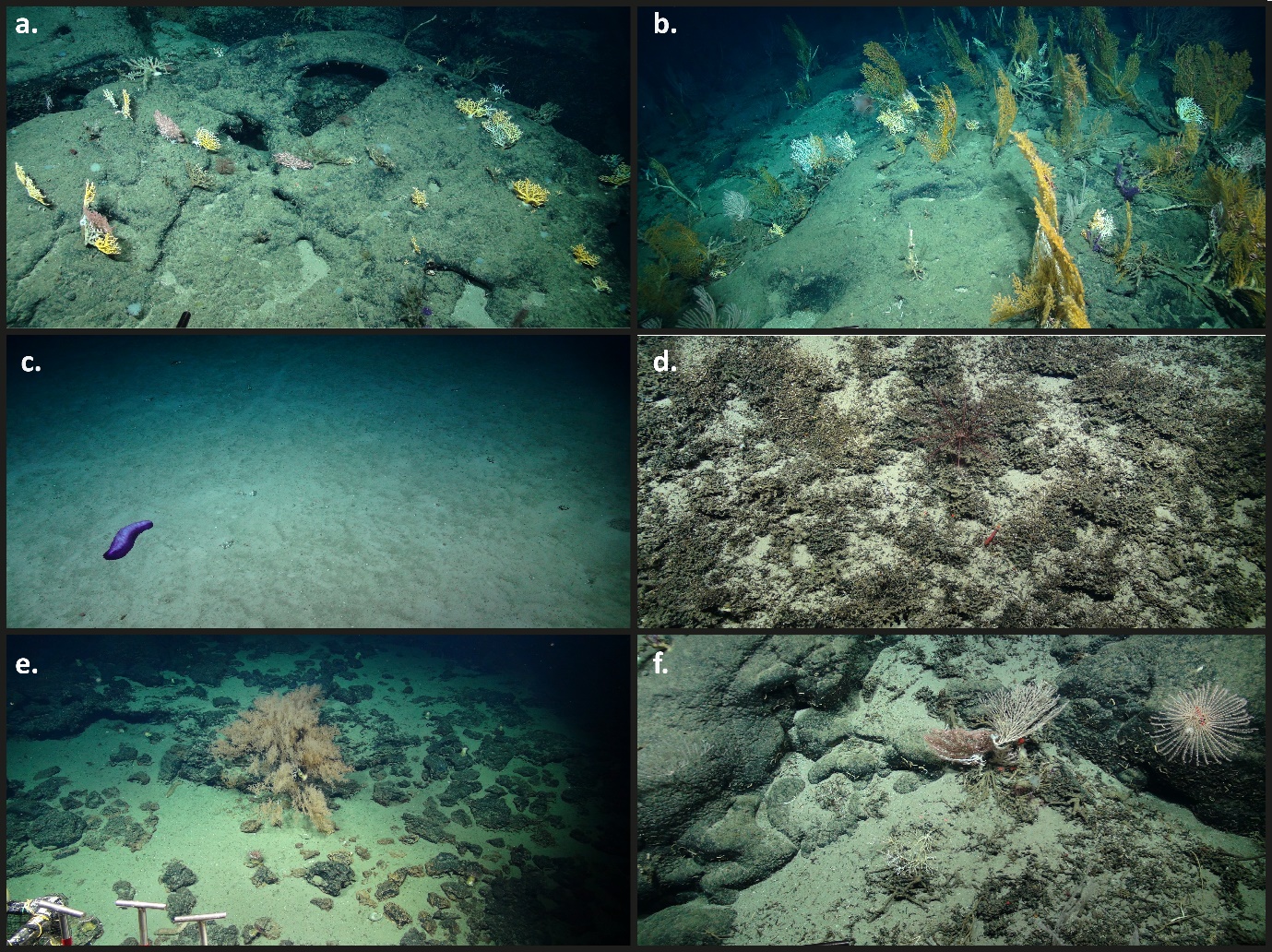


**Figure S1**. Example of the substrate classes used in the annotations from top left a. volcanic substrate with cold-water corals *Corallium* sp. and *Enallopsamia* sp., b. volcanic and biogenic with a *Paramuricea* sp. dominated coral garden attached to dead coral skeleton, c. sediment with the holothurian *Benthodytes* sp., d. sediment and biogenic with a Atelecrinidae crinoid, e. volcanic and sediment with the black coral *Leiopathes* sp. and holothurian *Stichopotidae* sp., f. volcanic, sediment and biogenic with cold-water corals *Corallium* sp. and *Iridogorgia* sp.

*.*
